# DEW: A wavelet approach of rare sound event detection

**DOI:** 10.1371/journal.pone.0300444

**Published:** 2024-03-28

**Authors:** Sania Gul, Muhammad Salman Khan, Ata Ur-Rehman

**Affiliations:** 1 Department of Electrical Engineering, University of Engineering and Technology, Peshawar, Pakistan; 2 Intelligent Information Processing Lab, National Center of Artificial Intelligence, University of Engineering and Technology, Peshawar, Pakistan; 3 Department of Electrical Engineering, College of Engineering, Qatar University, Doha, Qatar; 4 Department of Electrical Engineering (MCS), NUST, Islamabad, Pakistan; Air University, PAKISTAN

## Abstract

This paper presents a novel sound event detection (SED) system for rare events occurring in an open environment. Wavelet multiresolution analysis (MRA) is used to decompose the input audio clip of 30 seconds into five levels. Wavelet denoising is then applied on the third and fifth levels of MRA to filter out the background. Significant transitions, which may represent the onset of a rare event, are then estimated in these two levels by combining the peak-finding algorithm with the K-medoids clustering algorithm. The small portions of one-second duration, called ‘chunks’ are cropped from the input audio signal corresponding to the estimated locations of the significant transitions. Features from these chunks are extracted by the wavelet scattering network (WSN) and are given as input to a support vector machine (SVM) classifier, which classifies them. The proposed SED framework produces an error rate comparable to the SED systems based on convolutional neural network (CNN) architecture. Also, the proposed algorithm is computationally efficient and lightweight as compared to deep learning models, as it has no learnable parameter. It requires only a single epoch of training, which is 5, 10, 200, and 600 times lesser than the models based on CNNs and deep neural networks (DNNs), CNN with long short-term memory (LSTM) network, convolutional recurrent neural network (CRNN), and CNN respectively. The proposed model neither requires concatenation with previous frames for anomaly detection nor any additional training data creation needed for other comparative deep learning models. It needs to check almost 360 times fewer chunks for the presence of rare events than the other baseline systems used for comparison in this paper. All these characteristics make the proposed system suitable for real-time applications on resource-limited devices.

## Introduction

Over the past few years, the rise in crime rate and the resulting requirement of stringent security at many public and private places e.g. banks, automatic teller machines (ATMs), bus stations, café, public transport, offices, shopping malls, homes, etc. has resulted in a large scale installation of security cameras to monitor the surroundings automatically and avoid any unpleasant situation. The recordings of these cameras are not only used for avoidance but also help in later investigations, in case any unusual event occurs. However, the use of visual monitoring may not always work due to any obstruction in the line of sight of the camera, large unlit areas at night, bad weather conditions, occlusions that occur at overcrowded places, or events occurring outside the field of view of the camera [1–3]. Also, some events are not easy to spot on a video: for instance, a gunshot, a person screaming, or a tire skidding, but they have a very distinctive audio signature [1]. For this reason, there is a growing interest in refining audio surveillance methods for more accurate event detection. In comparison with video surveillance, one of the main advantages of audio surveillance is that they are not affected by variations in illumination and are equally effective at both day and night timings [1]. Also, the inexpensive equipment of audio surveillance as compared to video cameras has favored their usage [4]. However, the problem of designing audio surveillance models in an open environment is very challenging as the event of interest is superimposed to a significant level with the background noise [1]. Other challenges include inherent acoustic variability of the sounds belonging to the same event class, overlapping (simultaneously occurring) sound events, environmental noise, variability in the acoustic characteristics of the background acoustic scene, and rarely occurring sound events [5].

Sound plays a key role in identifying a rare event, whether it’s the anomalous machine sound classification [6,7], environmental sound classification [2], surveillance at public places (e.g. railway stations [2], subway stations [8], public squares [3], roads [4,9–11] and homes [12]), anomalous health conditions [13,14] or management of cowsheds with low manpower support [15]. Many studies detecting and classifying rare sound events have been proposed and are still underway. They are using signal processing, machine learning, deep learning, or their various combinations. A few models (e.g. [16]) have also combined video event detection (VED) with sound event detection (SED), as SED gets over most of the limitations of VED discussed above. The purpose of SED systems is to temporally locate and classify the rare event present in an acoustic signal [5]. The classification step is commonly called ‘audio tagging’.

In our proposed SED system, wavelets are used both for anomalous event detection and classification in an open environment. Wavelet-based rare event detection offers unique advantages over time and frequency-domain techniques. It is found that the impulsive parts of the audio, caused by the occurrence of a rare acoustic event are better characterized by wavelet-based features [17]. In time domain techniques, it is difficult to resolve the peaks arising due to the occurrence of sudden sound events. The time-domain features (e.g. zero-crossing rate (ZCR), amplitude envelop, and root mean square energy) are extracted directly from the raw signal so they are easy to implement [18]. However, they are not preferred for non-stationary signals as the statistical properties of these signals change over time. Secondly, as these features are calculated from signal amplitude values, so any interference acquired through recording comes as another disadvantage for them [19]. However, they are useful for measuring onset time and usually complement the frequency domain features. Under low signal-to-noise ratios, the frequency domain features (e.g. Fourier transform (FT), and short-time Fourier transform (STFT)) provide differentiation between environmental noise and the desired signal embedded inside it [20]. However, these features have their own drawbacks e.g. the Fourier transform lacks localization ability and is computationally slow for singular (rare) points, resulting in the Gibbs effect. Gibbs effect is the oscillating artifacts at the points of discontinuities, when the discontinuous functions are approximated by a truncated Fourier series [21]. STFT, on the other hand, is limited by the trade-offs involved in time and frequency resolution [22]. Also, it is not stable against time-warping deformations [23]. Among the spectral features, the Mel frequency cepstrum (MFC) and the Mel frequency cepstral coefficients (MFCCs) have been used for rare event classification (e.g. in SED models of [16,24]), due to their logarithmic representation which results in better separation of different signals with similar frequency contents [25]. However, it was found that in the case of MFC, the high-frequency spectrogram coefficients are not stable to time-warping distortions [26]. The MFCCs stabilize them by averaging them over the Mel band, but this averaging operation results in information loss (e.g. vibratos and attacks) [26], which degrades the classification accuracy. Another reason for the loss of information is due to discarding the higher frequencies in MFCC, which makes the distinction between the signals with similar timbre difficult. To reduce this information loss, MFCCs are usually computed over a smaller window, which makes the extraction of large-scale features challenging, limiting the performance of sound classification. In contrast, there is no requirement for the duration of the analysis window in the wavelet scattering network (WSN). In WSN, the time scattering coefficients are cascaded with frequency scattering coefficients to generate a feature representation of the signal [19]. The wavelet scattering coefficients (WSC) offer a stable and invariant signal representation for classification, without the loss of information associated with the Mel transforms. The most striking feature of wavelets is their astonishing similarity with the physiological models of the cochlea and auditory pathways. From the application’s perspective, there exists a multiplicity of information at different time scales, e.g. pitch and timbre at the scale of milliseconds, rhythm of music and speech at the scale of seconds, and urban sounds at the scale of minutes and hours. While MFCCs are efficient local descriptors for intervals shorter than 25ms, they fail to capture large-scale structures e.g. timbral structures such as attacks, frequency and amplitude modulations, and interference in musical chords. The coefficients of the wavelet transform, on the other hand, are calculated over larger window sizes thus allowing these larger structures to be captured without loss of information [23]. The accuracy of the sound classification task is found to improve with WSC as compared to STFT and MFCC [25]. Another advantage of wavelet coefficients is their flexibility in choosing their order [26]. Another advantage of wavelet coefficients is their flexibility in choosing their order [26]. WSN is equivalent to a convolutional neural network (CNN) with multiple stages (equivalent to CNN layers). Each stage of WSN is formed by the cascade of wavelets, modulus nonlinearities, and low-pass filters. The output of one stage becomes the input of the next stage. The number of such cascaded stages is called the ‘order’ of WSN. The order is usually kept low, to achieve low computational complexity suitable for resource-limited devices [27]. Energy dissipates as the signal moves from one stage to another. Research shows that the energy of the 3rd-order coefficients can fall below 1%, making the 2nd-order WSN sufficient for most of the applications [27]. WSN enables derivation of low-variance features from the real-valued time-domain signals. These features are insensitive to the translations of the input on an invariance scale defined by the user and also stable against time-warping deformations [28].

For the classification of the features extracted by the WSN, a support vector machine (SVM) classifier is used in our proposed model. As compared to other machine learning classifiers, SVM offers higher accuracy and computation efficiency with better generalization and also it requires less memory as it uses only a subset of samples during the decision phase [29].

Wavelets have long been used to detect anomalies from auscultation signals e.g. in [13,14,30], and recently for the detection of other health anomalies e.g. abnormal blood sugar levels [31], arrhythmia [32] and examine the functional connectivity in different brain regions in electroencephalogram (EEG) [33]. For the physiological time series data, e.g. brain and heart signals which are typically non-stationary, the wavelet transform has been used over recent years as a powerful tool to manipulate such signals [33]. Apart from these slowly varying medical signals, wavelets have been used for other time domain signals e.g. speech denoising [34], and anomaly tagging in machine sounds [35,36]. As compared to other feature extraction methods (time and frequency domain), wavelets are more suitable for the analysis of transient signal changes and irregular data patterns, where impulses may occur at any instant [37].

*Recent work using wavelets for sounds recorded in open environment*:

In the case of an open environment, the features extracted by wavelets are found to be beneficial in improving the accuracy of acoustic scene classification (ASC) tasks. In [38,39], the Mel-frequency discrete wavelet coefficients (MFDWC) are used for ASC to extract features from an acoustic scene to be later classified by the SVM network. Similarly, the wavelet-based spectrograms (scalograms) offer better multi-resolution analysis as compared to MFCC due to its suitability in adjusting both temporal window length and the wide frequency range [30]. In [40], scalograms are used for extracting the sound features to be processed by a cascade network comprising a two-dimensional pre-trained CNN model and gated recurrent neural networks (GRNNs) with a highway network layer and a softmax layer for ASC. In [41], ASC is carried out by an ensemble classifier (consisting of two random sub-space classifiers), trained on the features extracted from audio signals by WSN. The outputs of the two classifiers are combined by using the mathematical formula whose parameters are determined by a genetic algorithm. In the case of environmental sounds, the model of [23] uses WSN in fusion with the self-attention mechanism for ASC. In the ASC model of [23], WSN is used to extract features that are processed by the classical feed-forward neural network. The gunshot localization and classification model proposed in [42] uses wavelet MRA for denoising the acoustic signals contaminated by wind noise recorded by four microphones. The model then uses time-domain cross-correlation to localize the source and extreme learning machine (ELM) to classify the type of shot. A very recent work using WSN is a lightweight model [25] designed for the classification of infant baby cries. This system uses the features extracted by WSN and inputs them in a series of convolutional neural network (CNN) and residual blocks, where the CNN blocks provide the depth-wise and point-wise 2D convolutions to reduce the computational complexity during the training process, and residual blocks serve to strengthen the pattern recognition and avoid the gradient vanishing problem. However, this model only provides information about the presence of the rare event in an audio clip, without its time of occurrence. The model in [43] proposes the use of an SVM to cluster the features extracted from the audio by WSN for detecting any change in the ambient routine of elderly people. However, neither results are reported in their paper, nor any comparison is made with other models. The paper of [32] uses scalograms to fine-tune a pre-trained image classification network for the bird’s song classification. The use of pre-trained networks requires less training time and samples. However, this network is only able to classify these rare events but unable to detect their onset time.

***Contribution of this paper*:** The main contribution of our work can be summarized as follows:

1. Although wavelets have been used for denoising the recordings of the open environment for rare event detection (e.g. [42]) or its tagging (e.g. [23,25,32,38,39,41]) but not simultaneously for both purposes. In this study, to the best of our knowledge, this is the first time that wavelets are being used both for the rare event detection and its classification, for the events occurring in the open environment.
2. Unlike the models [42] (for gunshot) and [25] (for baby cry), which can classify only a single type of acoustic event, our proposed model is trained and tested for three types of acoustic events namely gunshot, glass break, and baby cry. Also, these models need additional deep learning modules (for example CNN and residual blocks in [25] and ELM in [42]), which results in an increased number of learnable parameters and computational cost as well as longer training duration and datasets, as opposed to our proposed network which has no learnable parameters and so it is faster and computationally more efficient. Also, the models [25,42] have been tested in only one kind of background noise (i.e. wind and domestic environment respectively), our proposed model is tested for 15 different kinds of background noises.
3. Although the model of [43] is designed for SED and is very much similar to our proposed system in using the WSN for feature extraction and later SVM for their classification, the main difference lies in the fact that their model uses no denoising mechanism before extracting features by WSN. Also, as they have not reported their results and have trained their model on their self-recorded dataset, it is not possible to compare our proposed algorithm with [43].

The rest of the paper is organized as follows. In next section, an overview of our proposed system is presented. After that, the experimental setup, the evaluation criteria, and the brief introduction of the comparison algorithms are given. The experimental results are given in the ‘experiment and results’ section and the conclusions are drawn in the last section.

## Proposed methodology

Our proposed SED system is named ‘DEW’, the acronym for ‘**D**etection of rare **E**vents by **W**avelets”. In the discussion below, the important steps of DEW are described.

### Feature extraction and support vector machine (SVM) classifier’s training

Before processing the signal by DEW, its SVM classifier needs to be trained. WSN is used to extract the important features from audio clips of the ‘anomalous’ or ‘rare event’ class(es) and the ‘background’ class to train our SVM network. All the notations and equations used in this subsection are adopted from [44]. Let *f*(t) be a 1-second audio sample of the training dataset for the SVM classifier. It may belong to any of the rare event classes (baby cry, glass break, gunshot) or the background class. The low-pass filter *ϕ* and the wavelet function *ψ* are designed to build filters, which can cover the whole frequencies contained in the signal. Let *ϕ*_*J*_ (*t*) be the low-pass filter that provides local translation invariant descriptions of *f* at a predefined scale *T*. The family of wavelet indices is denoted by *Λ*_*k*_, having an octave frequency resolution *Q*_*k*_. The multi-scale high-pass filter banks ${\left\{\psi_{j_{k}}\right\}}_{j_{k}\in \Lambda_{k}}$ can be constructed by dilating the wavelet *ψ*.

WSN iterates over three operations: 1) wavelet transform, 2) nonlinear modulus, and 3) averaging operation. The convolution $S_{0f(t)}=f*\phi_{J}(t)$ generates a local translation invariant feature set of *f*, but also results in the loss of high frequency information. These lost high frequencies can be recovered by a wavelet modulus transform

$$|W_{1}(f)|={\left\{S_{0f(t)},|f*\psi_{j1}(t)|\right\}}_{j_{1}\in \Lambda_{1}}$$ (1)

where ‘*’ shows the convolution operator. The first-order scattering coefficients are obtained by averaging the wavelet modulus coefficients with *ϕ*_*J*_ as:

$$S_{1f(t)}={\left\{|f*\psi_{j1}(t)|*\phi_{J}(t)\right\}}_{j_{1}\in \Lambda_{1}}$$ (2)

To recover the information lost by averaging, note that *S*_1*f*(*t*)_ is a low-frequency component of |f*ψ_*j*1_(*t*)|, from which the complementary high-frequency coefficients are extracted as given in Eq (3)

$$|W_{2}||f*\psi_{j1}|={\left\{S_{1f(t)},|\left|f*\psi_{j1}|*\psi_{j2}(t\right)|\right\}}_{j_{2}\in \Lambda_{2}}$$ (3)

Following the same pattern, the second-order scattering coefficients are defined as:

$$S_{2f(t)}={\left\{|\left|f*\psi_{j1}|*\psi_{j2}(t\right)|*\phi_{J}(t)\right\}}_{j_{i}\in \Lambda_{i}}$$ (4)

Iterating the above process defines the wavelet modulus convolutions *U*_*mf*(*t*)_ as:

$$U_{mf(t)}={\left\{\left|\left|f*\psi_{j1}|*\ldots \ldots .\psi_{jm}(t\right)\right|\right\}}_{j_{i}\in \Lambda_{i}},\;i=1,2,\ldots \ldots ..m$$ (5)

Averaging *U*_*mf*(*t*)_ with *ϕ*_*J*_ gives the *m*^*th*^-order scattering coefficients *S*_*mf*(t)_

$$S_{mf(t)}={\left\{|\left||f*\psi_{j1}|*\ldots ..|*\psi_{jm}(t\right)|*\phi_{J}(t)\right\}}_{j_{i}\in \Lambda_{i}},\;i=1,2,\ldots \ldots ..m$$ (6)

The final scattering matrix ***S***_***f***(***t***)_ aggregates scattering coefficients of all orders to describe the features of input signal as given in Eq (7):

$$S_{f(t)}={\left\{S_{mf(t)}\right\}}_{0\leq m\leq l},$$ (7)

where *l*′ is the maximal decomposition order.

The WSN is invariant to translations up to the invariance scale, which can be potentially large, due to the average operation determined by the low-pass filter *ϕ*_*J*_. The invariance scale establishes the time-scale of the low-pass filter and hence plays an important role in audio classification tasks [25]. Due to the properties inherited from wavelet transform, the features ***S***_***f***(***t***)_ are stable to local deformations. The structure of a WSN is similar to the convolutional neural network (CNN), but there are two major differences: 1) the filters are not learned but are set in advance and 2) the features are not only the output of the last convolution layer but also the combination of all those layers. The energy of scattering coefficients decreases rapidly as the layer level increases, with almost 99% of the energy contained in the first two layers [45]. Therefore, most of the networks, including ours, are confined to second-order to extract important features of audio signals. This also results in a significant reduction in computational complexity.

**Fig 1:**
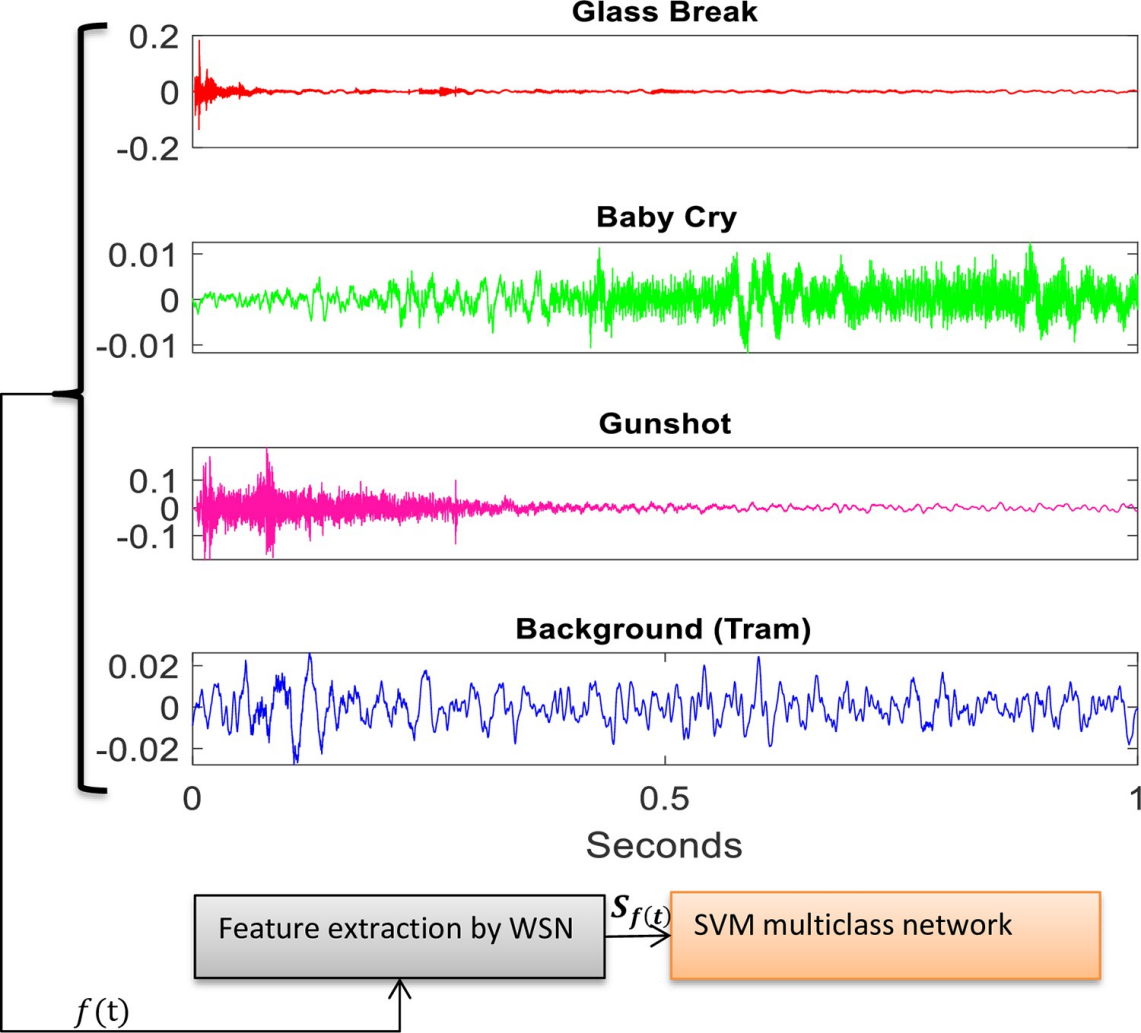
Audio samples of different classes, used for SVM training.

The extracted features are then used for training a multiclass SVM classifier. The SVM classifier is configured in one-against-all (OAA) mode. This mode of SVM constructs one SVM model per class, which is trained to discriminate the samples of one class from the samples of all other remaining classes. In OAA mode, the overall classification is achieved by using majority voting, where each SVM model votes for one class [35]. It is found that the OAA approach produces higher accuracy than the one-against-one (OAO) method but its training and testing take longer [46]. One sample of an audio clip from each class, used for the training of the SVM classifier is shown in the time domain as in Fig 1. The trained SVM is then inserted in the DEW network to do the audio tagging.

#### DEW testing

**Fig 2:**
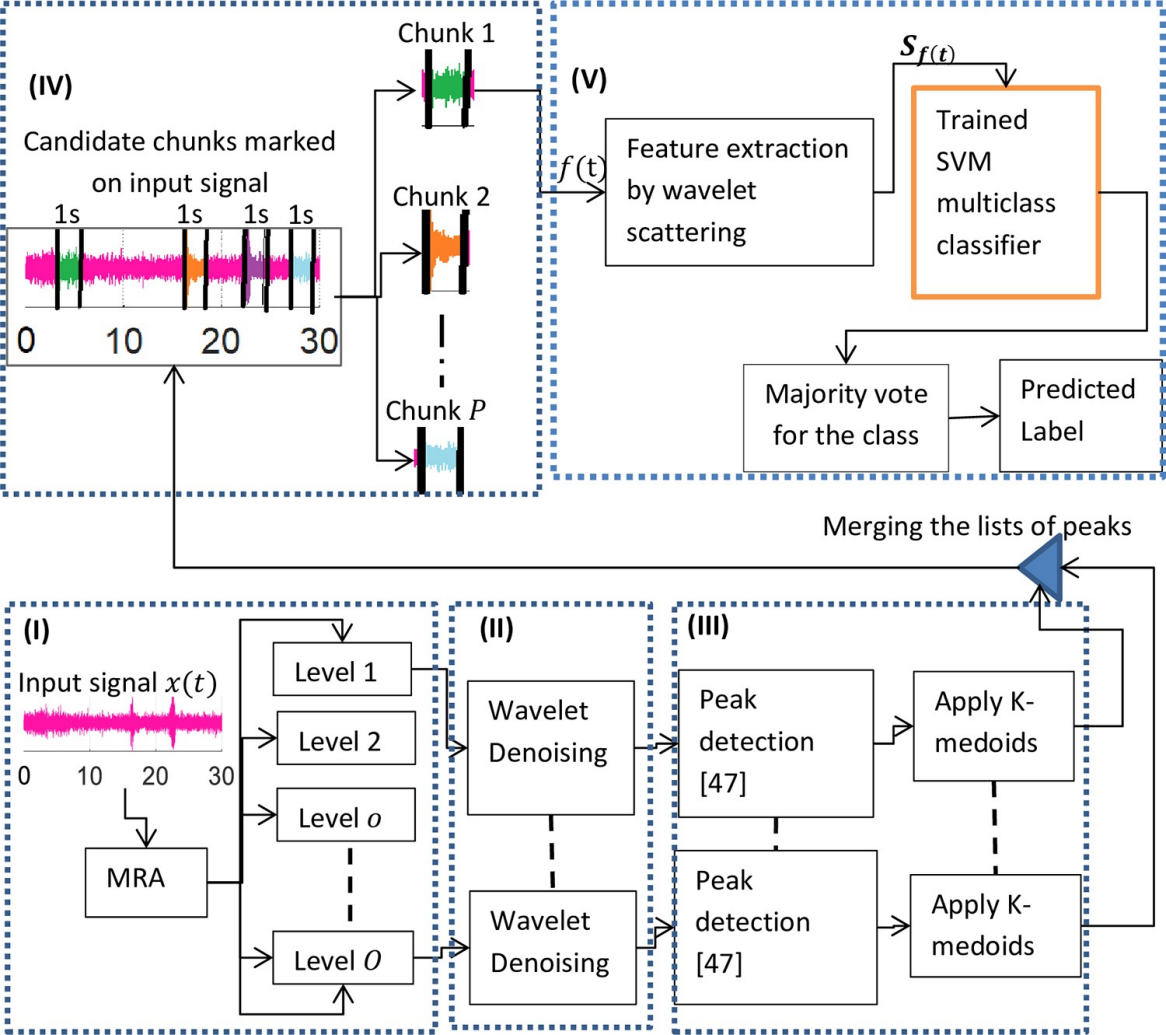
Signal flow inside DEW.

After the insertion of a trained SVM classifier, the signal flow diagram of DEW during the testing phase is shown in Fig 2. The boldfaced Roman numbers (enclosed in parenthesis) inside each dotted sub-block show the important steps involved. The audio signal (containing the rare event) is processed according to the sequence of steps given in this figure. Eqs (8) to (11) used in this subsection are adopted from [34] and Eqs (12) and (13) from [47].

***Step (I)*:** Multiresolution analysis (MRA) of an audio clip

The input audio clip, represented by *x*(*t*), has two main components, the rare event *r*(*t*) and the background *b*(*t*), as given by Eq (8).

x(t)=r(t)+b(t) (8)

where *t* is the sampling time.

This input signal *x*(*t*) is decomposed into multiple levels by using discrete wavelet transform (DWT) by the formula given in [34] as:

$$DWTx(a,b)=\int_{-\infty }^{\infty }x(t)\psi^{*}(t)$$ (9)

Where $\psi^{*}(t)=\psi_{m,n}(t)=2^{-m}\psi (2^{m}t-n)$ is the dilated and translated version of mother wavelet *ψ*, *a* = 2^−*m*^, *b* = *n*2^−*m*^ and *m*, *n*∈*Z*^+^.

**Fig 3:**
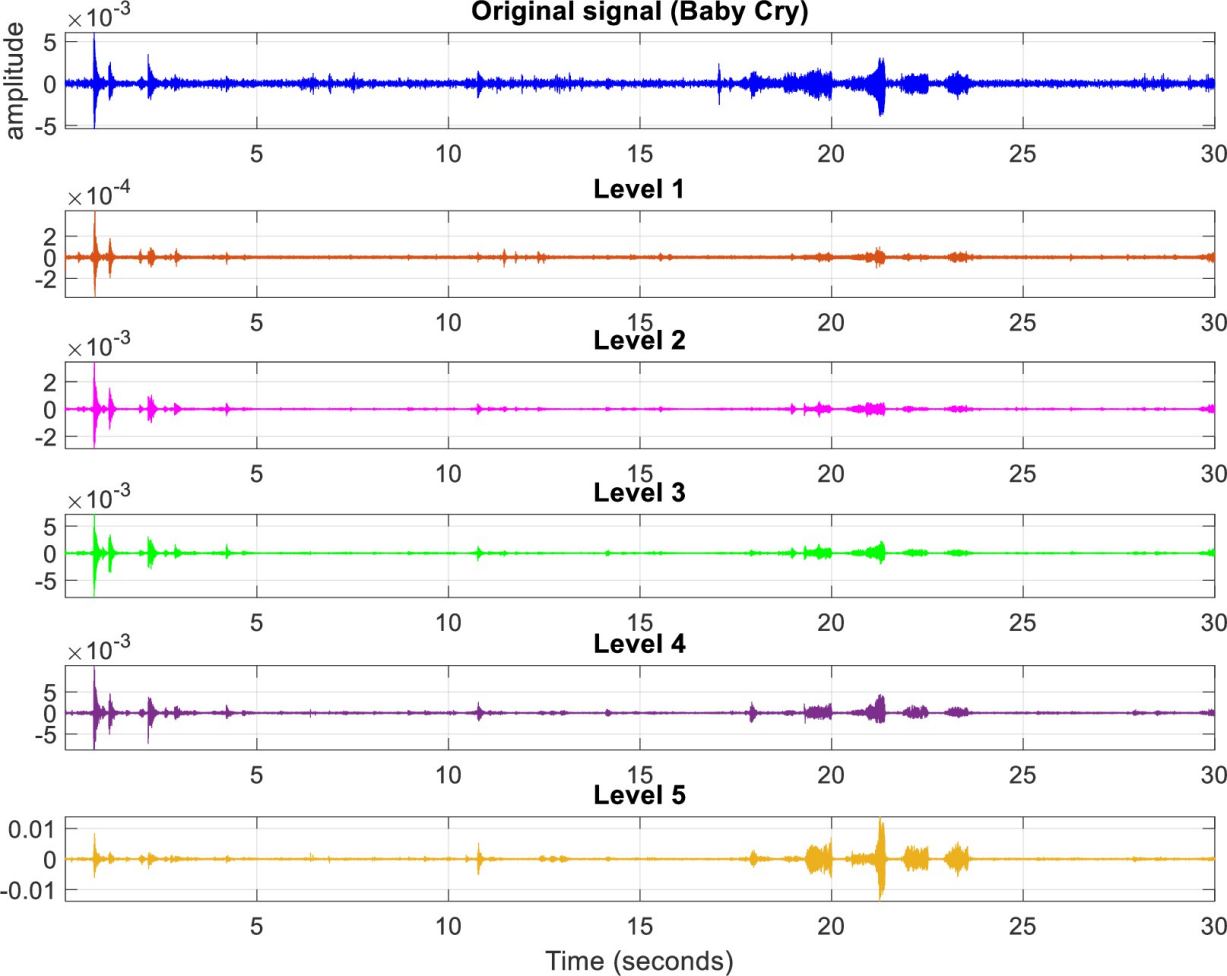
Audio clip decomposition into five levels by DWT.

With this choice, there exists a multiresolution analysis (MRA) algorithm, which decomposes the signal into scales with different time and frequency resolutions. MRA refers to breaking up a signal into components, which can produce the original signal exactly, when added back together. The term MRA is often associated with wavelets or wavelet packets, but there are non-wavelet techniques that also produce useful MRAs. Real-world signals are composed of many components. Often, only the first few are enough [22]. MRA allows us to narrow down our analysis by separating the signal into components at different resolutions. MRA provides a way of avoiding the need for time-frequency analysis while allowing us to work directly in the time domain. MRA can help localize and detect transient features like impulsive events. These changes are more easily visualized in the components of MRA than in the raw data [48]. The wavelet decomposition results in multiple levels of approximated and detailed coefficients. Let’s represent each level by *level*_*o*_, where *o* = 1,2,……….*O*. The decomposition of *x*(*t*) into *O* levels (where *O* = 5 in our case) is shown in Fig 3.

DWT decomposes the signal by convoluting it with the coefficients of high-pass and low-pass filters. These two filters are quadrature mirror filters (QMF) [49]. The outputs of a low-pass filter are known as the approximated coefficients, and the results of high-pass filters are known as the detailed coefficients. The DWT begins by finding the approximated and detailed coefficients from the input signal. For higher decomposition levels, the approximation coefficients are further filtered at every layer into approximated and detailed coefficients following dyadic sampling. A wavelet basis function must be able to decompose and reconstruct the signals efficiently. Orthogonal wavelet basis functions are found to have such characteristics, so they can be a good choice for anomaly detection.

***Step (II)*:** Denoising the MRA levels

In the class of adaptive filters, wavelet-based denoising has shown promising results [42]. The levels obtained from MRA get sparser as one goes up the levels, keeping only the most important signal details (transitions). Each level *o* has a different number of samples *N*. However, the signal information is embedded in noise (background signal *b*(*t*)), and so it is necessary to denoise these levels to retrieve the embedded information. Let *W*(.) denote the forward wavelet transform operator and *D*(.,*λ*) the denoising operator with threshold *λ*. The two steps involved in the denoising process of *level*_*o*_ by wavelets are given as in [34] by Eqs (10) and (11):

$$Y=W({level}_{o})$$ (10)

$$Z=D({level}_{o},\lambda )$$ (11)

There are four common rules for selecting the threshold *λ*. These are 1) ‘heursure’, 2) ‘minimax’, 3) ‘rigsure’ and 4) ‘sqtwolog’. The first two of them are more conservative and would be more convenient when small details of the signal lie near the noise range. The last two of them remove the noise more efficiently. The threshold *λ* is either dependent only on the number of samples *N* (e.g. in minimax) or it can be data-adaptive (e.g. in Stein’s Unbiased Risk Estimator (SURE)), which depends not just on *N* but also on data. There are two types of thresholding; 1) hard and 2) soft. In hard thresholding, the elements whose absolute values are less than *λ*, are set to zero. In soft thresholding all those elements whose absolute values are less than *λ* are set to zero, while those above it are shrunk towards 0. Hard thresholding is the simplest method and provides edge preservation, but the soft one has nice mathematical properties [34] and provides smoother results [50]. The package of [51], implementing the soft thresholding, is used for level denoising.

***Step (III)*:** Peaks detection

The rare events are usually impulsive and result in a transitional increase in the energy of surroundings. These transitions are well captured by the peaks of the decomposed levels of DWT. Not all peaks are necessarily linked with the onset of an event. However, some of them may contain useful information about the onset time of rare events. To detect them, the negative portion of each denoised level is clipped off and the peak finder algorithm of [47] is applied, where the threshold above the surrounding peaks is kept large for the algorithm to be more selective in finding the peaks. The clipping-off process on any level *level*_*o*_ and the threshold value ȿ set in the peak finder algorithm [47] are given by Eqs (12) and (13) respectively.


$${level}_{o}=\max\left(0,{level}_{o}\right)$$ (12)

$$ȿ=\frac{\max\left({level}_{o}\right)-\min\left({level}_{o}\right)}{2}$$ (13)

**Fig 4:**
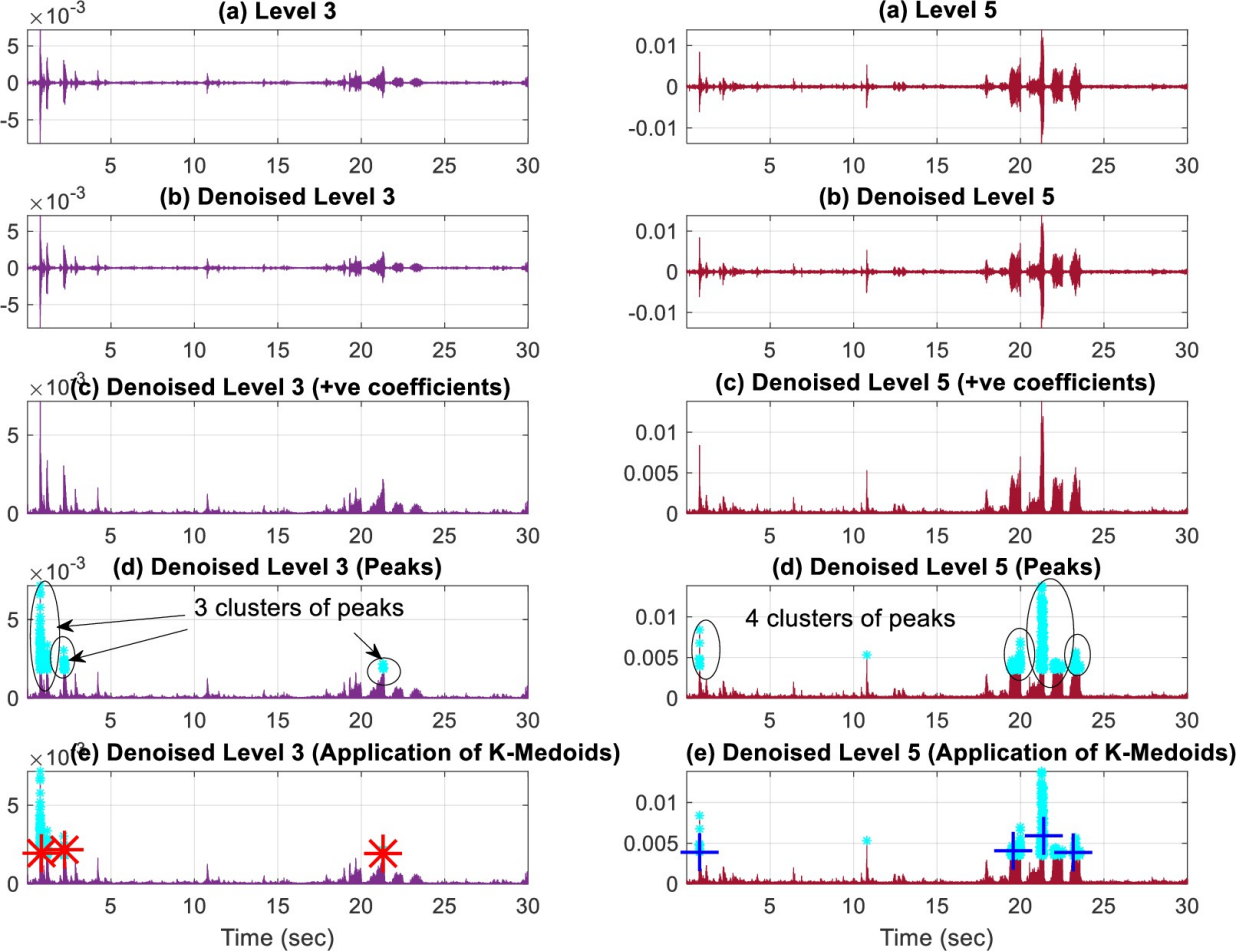
Peak finding on the 3^rd^ and 5^th^ level.

However, the algorithm [47] has resulted in producing many peaks in the surroundings of an actual peak, even after keeping the threshold ȿ to a very high value, due to the very high sampling rate of the recorded clips. To overcome this problem, the K-medoids algorithm is applied to narrow down the peak search to only a few prominent peaks. Although there are other unsupervised clustering algorithms e.g. density-based spatial clustering of applications with noise (DBSCAN) and spectral clustering to find the core points of clusters, but as they are computationally more expensive than the K-medoids, they are not considered here for solving this problem. To overcome this problem, the K-medoids algorithm is applied to narrow down the peak search to only a few prominent peaks. Although there are other unsupervised clustering algorithms e.g. density-based spatial clustering of applications with noise (DBSCAN) and spectral clustering to find the core points of clusters, but as they are computationally more expensive than the K-medoids, they are not considered here for solving this problem. Also, the K-medoids clustering offers lower execution time, reduced cluster overlapping, and improved clusters due to selecting a representative object instead of a non-representative object as done in K-mean clustering where the mean value of cluster is taken as its centre. The K-medoids clustering is also more robust to noise and outliers than the K-means clustering [52]. For K-medoids, the number of clusters that exist in each level must be known beforehand, which of course varies according to the audio clip and the MRA level under consideration. As the number of clusters cannot be set apriori in K-medoids, the algorithm of data partitioning for spectral clustering [53] is used, to find the appropriate number of clusters for the K-medoids algorithm. This results in reducing the total number of peaks to only a few prominent ones. As all MRA levels do not generate the peaks that are representative of the occurrence of rare events, only those levels are selected which help in the detection of rare events and others are discarded. Other constraints for selecting the optimal MRA levels will be discussed in the ‘experiment and results’ section. The denoising and peak detection operations are applied only on these selected levels to save computational resources and time. The process of peak finding for the 3^rd^ and 5^th^ denoised MRA levels is shown in Fig 4.

***Step (IV)*:** Marking and cropping the audio clip

**Fig 5:**
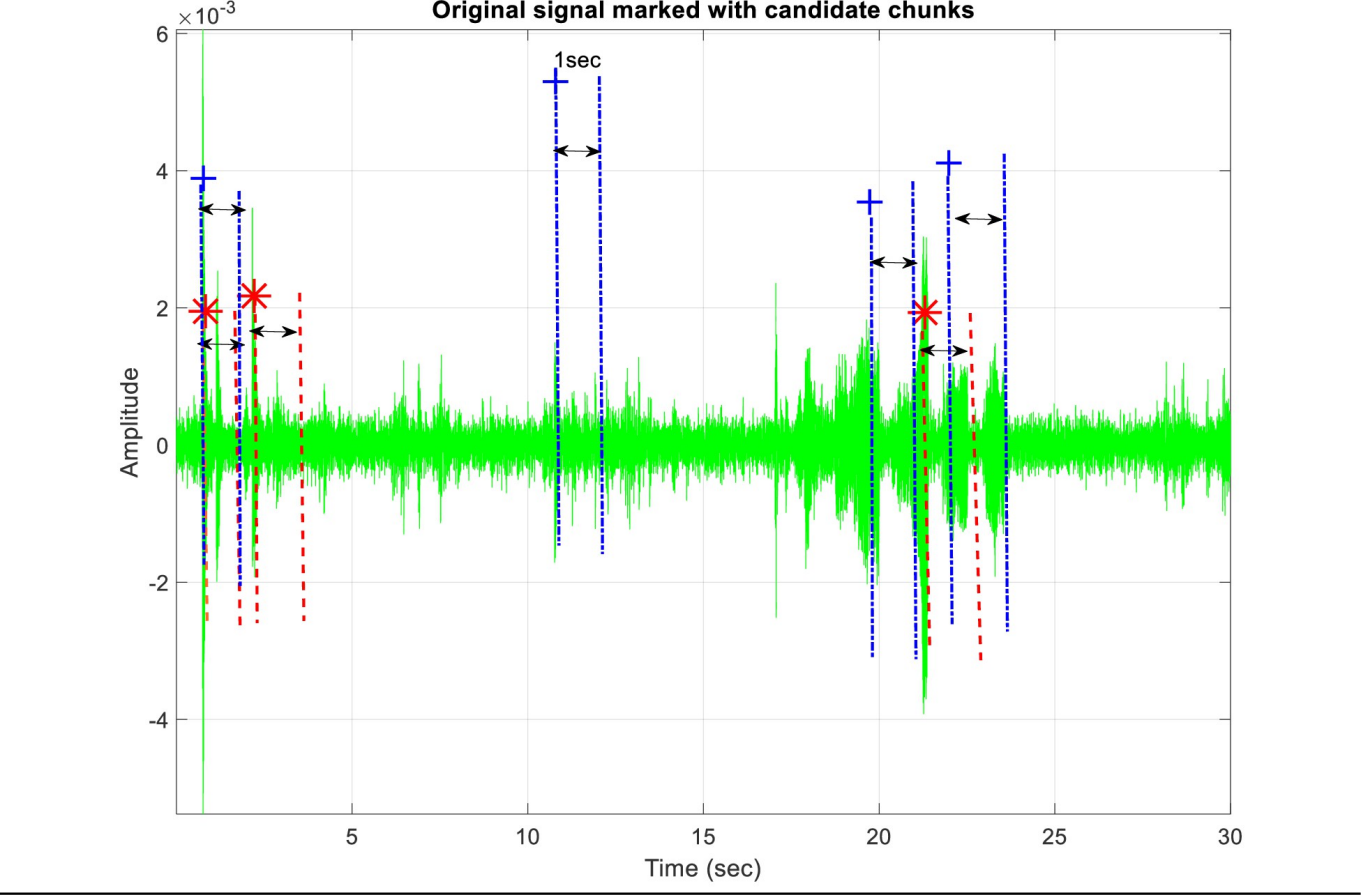
Peak marking from 3^rd^ and 5^th^ levels on original signal. ‘*’ shows the peaks detected by 3^rd^ level and ‘+’ shows the peaks detected 5^th^ by level.

After finding peak locations, only on selected levels of MRA, by step (III), these locations are converted to the time domain, their list is merged, and they are marked on our original time domain signal *x*(*t*) as shown in Fig 5. The merging of lists is not required in case a single MRA level is selected.

The location of these peaks over the original signal may indicate the onset of a rare event and so, they must be checked for its presence. The original signal *x*(*t*) is cropped into chunks of one-second duration, where each chunk will begin at one of the marked peak points as shown in Fig 5.

As many chunks are generated by cropping the input signal *x*(*t*) as there are peaks marked on the original signal. Assume there are total ‘*P*’ chunks generated from an audio clip of 30 seconds. The value of ‘*P*’ is not fixed for all clips and varies according to the number of peaks detected by step III. However, the duration of these chunks is fixed. All these one-sec duration chunks, each starting at one of the marked peak locations, are called the ‘candidate chunks’, from which the features are extracted by WSN to check for the presence or otherwise of the anomaly.

***Step V*:** Chunk tagging

The features from each of the ‘*P*’ candidate chunks are extracted by the WSN as described in subsection ‘feature extraction and support vector machine (SVM) classifier’s training’ and then passed to the trained multiclass SVM classifier to classify each chunk as either an ‘event’ or ‘background’, according to the majority voting rule.

## Experimental evaluation parameters

This section includes the dataset, metrics, and parameter settings for different component networks and a brief description of the comparative algorithms used in this paper to evaluate and compare the performance of our proposed SED model DEW with other models.

### Dataset

The dataset used for training and evaluation of DEW is the detection and classification of acoustic scenes and events (DCASE) 2017 challenge, task 2: “Detection of rare sound events” [54]. The dataset is composed of 3 classes i.e. baby cry, glass break, and gunshot. Each audio sample is 30 seconds in duration. The rare events in these clips are already mixed with any of the 15 different backgrounds, including beach, bus, café, car, city center, forest path, grocery store, home, library, metro station, office, park, residential area, train, and tram. The mixing is done at the event-to-background ratio (EBR) of 0, +6 dB, and -6 dB, resulting in 500 audio clips belonging to each of the three categories of baby cry, glass break, and gunshot respectively. Half of these clips have only the background sound and the remaining half contains the rare event mixed with background. In each audio clip, the rare event occurs only once during the entire 30-second duration, however at variable time instants. The sampling frequency of each audio file is 44.1 kHz. A Meta file is accompanied by the training data, containing the presence or otherwise of the rare event, its starting time, duration, EBR, and the background with which it is mixed in the clip. The evaluation dataset, however, is not accompanied by any Meta information. Also, it was found that the clips in the evaluation dataset are picked randomly from the training dataset, so testing DEW on the entire training dataset ensures that the evaluation dataset is included in the testing phase.

First, the dataset is divided according to the classes (baby cry, glass break, or gunshot) and then further according to the presence or absence of a rare event. After the split, 6 folders are created, containing 250 files each. Three of them have audio clips, containing the rare events belonging respectively to any one of the 3 classes: baby cry; glass break, and gunshot, mixed with different background types, and the remaining three folders contain only the backgrounds without the rare event. In the second data split, the samples in the last three folders (audio having only background without rare events) are first combined and then divided into 15 different folders, each belonging to one category of background.

For each background, a separate SVM classifier is trained. Each SVM network is trained on 4 classes i.e. 1) baby cry, 2) glass break, 3) gunshot, and 4) a particular background as shown in Fig 1. A total of 250 samples of each class are used for training the classifier. From the first three folders obtained after the first data split, in which the rare event exists, 1-second clips are cropped according to the rare event onset information obtained from its associated Meta file to create the dataset for the three rare event classes required for SVM training. Although the duration of the rare event varies in each of the 30-second audio clips (as shown in Table 1), the cropping duration is kept fixed to one second to ensure a uniform length training dataset for the SVM classifier. So, in these 1-sec clips, the inclusion of background is inevitable in all those cases, where the duration of the rare event is shorter than one second. Also, the type of background varies in all those cases, where it is included. Despite the risk of including some background in the dataset of rare event classes and deteriorating its quality, which in turn would affect the classifier accuracy, the training samples for SVM training are not taken from their source (the freesound.org; used by DCASE for preparing the dataset [54]), as the recordings there may contain the silence zones. These zones are already removed in the DCASE dataset. Also, our network is required to be trained on acoustic variability (different EBRs) which of course is not labeled on the recordings of freesounds.org. Apart from these 1-sec chunks taken from the training dataset of DCASE, no audio clip (either entirely or any of its cropped portions) is used for the training of DEW.

**Table 1:** Duration of rare events in DCASE dataset.

Rare event duration in DCASE 2017 dataset	Maximum (sec)	Minimum (sec)	Mean (sec)
**Baby cry**	4.82	0.48	1.8
**Glass break**	2.16	0.24	0.8
**Gunshot**	2.28	0.2	0.9

The fourth class for the SVM classifier contains 250 samples of background of a single type. One background folder is chosen from the 15 folders, obtained after the second data split. From the clips in this folder, 250 random portions each of one-second duration are extracted for the training of the SVM classifier dedicated to that background.

For the evaluation of DEW, instead of using the evaluation dataset, the training dataset of DCASE is used for two main reasons. The first is that our system has not seen this complete 30-second dataset during the training phase, so it is not an artifice to use it. The second is that the accuracy of detection of the rare event onset time can be checked by its Meta file which is not available with the DCASE 2017 evaluation dataset.

### Evaluation metrics

For the evaluation and comparison of our model with other models, the metrics used are error rate (ER) and F1 score. These metrics are the standard metrics to be used for the evaluation of models designed for Task 2 of the DCASE 2017 challenge [54]. Both metrics are calculated on the event basis as defined in [55], using a collar of 500ms taking into account only the onset time of the rare event. The event-based metrics measure the onset/ offset detection capability of a system in detecting the correct temporal position of an event [56]. Event-based ER and F1 can be calculated in two ways: 1) using onset conditions only, and 2) using both onset and offset conditions. As our main concern is in the correct estimation of the onset time of the rare event, so in this paper, the ER and the F1 scores are calculated using only the onset conditions [56] according to the prescription of the DCASE 2017 challenge [54].

### Comparison algorithms

DEW is compared with five other rare event detection algorithms that have used the DCASE 2017 dataset. The first three of them i.e. [5,55,57] are among those that have participated and were ranked as the top three methods at the DCASE 2017 challenge, while the last one is the SED algorithm presented in [58]. A brief introduction to these models is given below.

In [55], the authors use 1D CNN to extract the features from each time-frequency (TF) unit of the 2D log amplitude Mel-spectrogram of an audio clip. These features are then given as an input to a unidirectional backward long short-term memory (LSTM) network, to determine the precise onset timings. CNN is used for the extraction of localized features while LSTM is used to extract the long and short-term temporal dependencies in the extracted features [59]. The backward LSTM is used because it is found that for the accurate detection of onset, the information after the onset is more important compared to the information before it [55]. The output of the LSTM layer is then input into a fully connected layer terminated by a single neuron with a sigmoid activation function, which gives the probability of the presence of the target event. In [5], a convolutional recurrent neural network (CRNN) is used for SED. The convolutional neural network (CNN) of CRNN is useful to overcome the problem of intra-class acoustic variability by using the max pooling operation and the shared weight connections, and the recurrent layers of CRNN are useful for extracting the long-term temporal context of an audio clip for accurate rare event localization. In [57] both CNN and deep neural network (DNN) are tested for SED. Two networks (i.e. two DNNs for a DNN-based system and two CNNs for a CNN-based system) are used for background rejection and classification respectively. It was found that the CNN-based network performs better for baby cry and the DNN-based system for the other two classes. Finally, the model of [58] uses a set of input features comprising log Mel coefficients, WSN coefficients, and their first-order derivatives, along with WSN coefficients filtered by a linear prediction error filter and their first-order derivatives given to a two-stage event detector, where each stage is composed of CNN. The first stage acts as a binary classifier which proposes a set of contiguous blocks to the second stage, which refines the classification of the first stage by discarding the blocks wrongly classified as containing rare events. Among the leftover contiguous frame chunks classified as “event”, the first frame from the left side generating the highest network output is regarded as the event onset time. In the SED model of [60], a CRNN-based temporal-frequential attention model is proposed focusing simultaneously on important frames and their frequency contents. The input features extracted from small chunks of audio are extracted by log-energy filter bank and given to CRNN having an architecture similar to the one used by the SED model [5] except that [5] uses an ensemble of networks which produce the lowest error rate results while [60] does not.

### Hyper-parameter settings

The hyper-parameters settings for the processes occurring in DEW are listed in Table 2.

**Table 2:** Hyper-parameter settings for different operations.

Process	Parameters	Values
MRA [51]	Mother wavelet	‘sym6’
	Detailed levels	5
	Number of coefficients in level 1	661501
	Number of coefficients in level 2	330751
	Number of coefficients in level 3	165376
	Number of coefficients in level 4	82688
	Number of coefficients in level 5	41344
**Wavelet denoising [51]**	Denoising method	‘rigsure’
	Type of thresholding	Soft
	Mother wavelet	‘sym6’
	Detailed levels	5
	Multiplicative threshold rescaling	‘mln’ (level-dependent estimation of noise)
**SVM classifier hyper parameters [61]**	Kernel function	Polynomial
	Polynomial order	2
	Kernel scale	Auto
	Box constraint	1
	Training samples per class	250
	Duration of each training sample	1 sec
	Training mode	5 folds cross validation
**Wavelet scattering network [61]**	Sampling frequency	44100 Hz
	Invariance scale	0.5
	Number of samples	44100 = 1 sec
	Number of scattering paths	300
	Number of coefficients for each scattering path	11
	Time windows in subsampled scattering framework	2
	Number of features extracted per time window	418
	Batch size	64

Hyper-parameter optimization is the search for the set of hyper-parameter values that achieves the best performance on a given task in a reasonable amount of time [62]. Normally there are two main methods of hyper-parameter optimization 1) manual search and 2) automatic search algorithms e.g. grid search method, genetic algorithm, and Bayesian optimizer. Manual search requires background knowledge and is difficult for non-expert users. In the hyper-parameter settings for different modules, a manual search method is adopted for finding the number of MRA decomposition levels required by DEW. The model in [22] discusses the importance of hyper-parameter tuning of discrete wavelet transform used to extract the features from the audio which were then used as input to an artificial neural network. They suggest that the wavelet decomposition level must not exceed 2, but as our model fails to detect a large number of rare events with a decomposition level setting of 2, the decomposition levels are gradually increased from 1 to 5 and finally good performance is achieved with 5 levels. Except for the ‘coiflets’ and ‘reversebior’ families where the accuracy of detection is very low for high levels, it is shown in [22] that all wavelet families give a similar performance for high decomposition level settings. So, the ‘symlets’ family is used in DEW as in [51]. All other wavelet denoising parameters are kept the same as in [51] except the multiplicative threshold level which is ‘mln’ instead of ‘sln’ as a nonwhite noise is suspected, and so the thresholds must be rescaled by a level-dependent estimation of the level noise [63].

The dataset of 1s audio clips, belonging to three “rare-event” classes and a single ‘background’ class are stored in four folders (with class labels as their names). The number of samples is the same in each folder. The data inside each folder is shuffled randomly and split into two parts. 80% of data is reserved for training and 20% for testing. Then 5-fold cross-validation is used for training the SVM classifier. In the SVM classifier’s settings, two-time windows and a simple majority voting rule over these windows are used to assign the class label to an event. If there is no majority, the class "NoUniqueMode" is assigned and it is considered a classification error [61]. All hyper-parameters of the SVM classifier and WSN are adopted from [61]. Matlab supports three types of SVM kernels 1) Gaussian (radial basis function (RBF)) 2) linear and 3) polynomial. Gaussian and linear kernels do not apply to our problem as these kernels are meant respectively for one and two-class learning models [64] and in our case, the SVM classifier has to classify four class datasets (baby cry, glass break, gunshot, and background). So, a second-order polynomial kernel function is used for the SVM classifier of DEW.

As already stated above to reduce the computational load and enable the model implementation on a resource-limited device, WSN is limited to second-order in DEW. Also, to stabilize the network to local deformations (time translations and frequency transpositions); the invariance scale is set to 0.5 as in [61].

## Experimentation results and comparison

In this section, the performance of our proposed model DEW is evaluated and compared to other SED models.

***Case 1*:** Selecting the optimal levels of MRA

As shown in step (I) of the proposed methodology, MRA results in multiple levels. However, not all of them are useful for SED. In this experiment, each level is tested for three conditions; 1) its ability to detect the ‘rare event’, 2) its ability to reduce the computational cost, and 3) its precision in calculating the onset time of the event. The more these three conditions are fulfilled by any level, the more it becomes eligible for selection. There are 250 files, each of 30 seconds, containing the rare events in the 3 folders, obtained after the first data split (see subsection ‘Dataset’). MRA is used to decompose each audio clip into 5 levels as shown in Fig 3 and the candidate chunks are extracted from each clip according to the peaks marked on it according to these levels. However, the peak finding method described above fails to discover any peak in levels 1, 2, and 4 for most of the audio clips, resulting in the failure of SED on these levels. In this paper, this type of failure would be called a ‘Type 1’ failure. Apart from this failure, there is also another type of failure in the detection process, where the DEW fails to detect the peak within the given 500ms collar of the rare event onset time given in its associated record in the Meta file. Here this type of failure is called a ‘Type 2’ failure. In Type 2 error, peaks are detected but none of them has any useful information (rare event), as their locations are outside the predefined collar of 500ms of the event’s onset time. Both types (1 and 2) of these errors can be regarded as ‘detection failure’. The number of detection failures due to either type 1 or type 2 errors for all the MRA levels, along with the failure count breakdown according to the EBRs, is listed in Table 3 for all the rare event classes.

**Table 3:** Type 1 and 2 errors for MRA levels according to class and according to EBR.

Class	Level	1^st^			2^nd^				3^rd^				4^th^			5^th^				3^rd^ & 5^th^		
Baby cry	**Type 1 failure**	34			4				0				1			0				0		
	**Type 2 failure**	90			240				35				86			26				9		
	**EBR wise Type 1 & 2 failures**	**0 dB**		38	**0 dB**			79	**0 dB**			12	**0 dB**		25	**0 dB**	7			**0 dB**		2
		**+6 dB**		37	**+6 dB**			93	**+6 dB**			8	**+6 dB**		31	**+6 dB**	4			**+6 dB**		2
		**-6 dB**		49	**-6 dB**			72	**-6 dB**			15	**-6 dB**		31	**-6 dB**	15			**-6 dB**		5
	**Total**	124			244				35				87			26				9		
Glass break	**Type 1 failure**	4			3				0				16			0				0		
	**Type 2 failure**	5			239				23				25			71				20		
	**EBR wise Type 1 & 2 failures**	**0 dB**	3		**0 dB**	76			**0 dB**		4		**0 dB**	17		**0 dB**			27	**0 dB**	2	
		**+6 dB**	0		**+6 dB**	85			**+6 dB**		3		**+6 dB**	14		**+6 dB**			11	**+6 dB**	5	
		**-6 dB**	6		**-6 dB**	81			**-6 dB**		16		**-6 dB**	30		**-6 dB**			33	**-6 dB**	13	
	**Total**	9			242				23				61			71				20		
Gunshot	**Type 1 failure**	14			2				0				3			1				1		
	**Type 2 failure**	14			242				37				52			42				20		
	**EBR wise Type 1 & 2 failures**	**0 dB**		8	**0 dB**		86		**0 dB**	12			**0 dB**		19	**0 dB**		13		**0 dB**	10	
		**+6 dB**		7	**+6 dB**		75		**+6 dB**	7			**+6 dB**		10	**+6 dB**		5		**+6 dB**	2	
		**-6 dB**		13	**-6 dB**		83		**-6 dB**	18			**-6 dB**		26	**-6 dB**		25		**-6 dB**	9	
	**Total**	28			244				27				55			43				21		
**Type 1 & 2 failures**	**Failure rate**	0.214			0.973				0.113				0.271			0.186				0.07		

As compared to other levels, very few type 1 and type 2 errors occur at the 3^rd^ and 5^th^ MRA levels and this count is lowest when both of them are combined. This combination results in the reduction of type 2 errors, as the rare events missed by the 3^rd^ level are detected by the 5^th^ level and vice versa. The failure rate is the ratio of the total number of audio clips failing the detection test to the total number of audio clips containing rare events (750 in our case). It is lowest when the 3^rd^ and 5^th^ levels are combined. At any individual level, most of the detection failures occurred at the EBR of -6dB. Event detection by the merger of the 3^rd^ and 5^th^ levels benefits this ERB the most. So our proposed algorithm is checked only at the 3^rd^ and 5^th^ levels and at their combination.

Apart from the detection failure, the computational cost and the precision of event onset timings generated by any level are also checked. For this, the total number of candidate chunks *P* generated by each decomposed level (or by their combination e.g. by combining 3^rd^ and 5^th^ levels) is calculated. As there is a single event in each audio clip of 30 seconds, so more the candidate chunks generated for an audio clip, the more time would be required to check them one by one for the presence of an event. If multiple chunks are tagged as ‘rare event’ chunks, the winning chunk, among all the *P* candidates, would be the one, whose onset time is the closest match to the time given in the Meta file. For all MRA levels, the average deviation of the ‘winning chunk’ from its Meta file timings is given in Table 4. Note that these values are calculated for the audio clips that do not fall victim to detection failure test (Type 2 errors).

**Table 4:** Average number of candidate chunks generated and the average onset time deviation of the winning chunk for each MRA level.

Class	Level	1^st^	2^nd^	3^rd^	4^th^	5^th^	3^rd^ & 5^th^
**Baby cry**	*P*	2.59	2.6	4.2	**2.4**	3.52	7.7
	Average onset deviation of the winning chunk from its Meta file time (sec)	0.572	**0.489**	0.543	0.67	0.6	0.5
**Glass break**	*P*	**1.2**	1.46	2.996	1.78	3.5	6.57
	Average onset deviation of the winning chunk from its Meta file time (sec)	0.06	0.19	0.09	**0.05**	0.14	0.12
**Gunshot**	*P*	**1.61**	2.03	3.98	1.94	3.35	7.33
	Average onset deviation of the winning chunk from its Meta file time (sec)	0.13	0.45	0.092	**0.075**	0.16	0.125

The lowest values for each class are boldfaced. Although the value *P* and the average onset time deviation at the 1^st^, 2^nd^, and 4^th^ levels are the lowest for various classes, they are not preferred due to their very high detection failure rate.

In the next three experiments, the performance of DEW is evaluated at the 3^rd^ and 5^th^ level and at the combination of these two levels.

***Case 2*:** Evaluation of DEW at 3^rd^ level

Table 5 shows the performance of DEW for baby cry, glass break and gunshot in 15 different backgrounds at the 3^rd^ decomposed level.

**Table 5:** 3^rd^ level results.

Background	Baby cry		Glass break		Gun shot	
	ER	F1 (%)	ER	F1 (%)	ER	F1 (%)
**Beach**	0.42	64.28	0.05	96.97	0.29	81.48
**Bus**	0.23	86.67	0	100	0.27	84.2
**Café**	0.56	60.87	0.267	84.61	0.21	86.95
**Car**	0.34	80	0.095	95	0.23	88.23
**City-Centre**	0.35	75.86	0.133	92.85	0.21	86.95
**Forest-Path**	0.31	76.19	0.71	62.22	0.52	56
**Grocery-Store**	0.45	61.54	0.1	94.74	0.47	63.15
**Home**	0.37	53.334	0.09	80	0.4	63.63
**Library**	0.15	88.9	0.05	96.55	0.125	90
**Metro-Station**	0.18	83.34	0.22	86.66	0.25	84.61
**Office**	0.3	75	0.053	96.97	0.23	82.76
**Park**	0.5	64.28	0.06	96.29	0.36	78.26
**Residential-Area**	0.35	72	0	100	0.21	85.7
**Train**	0.59	58.33	0.1	94.74	0.25	85.7
**Tram**	0.6	57.14	0.04	97.67	0.14	91.67
**Average**	0.38	70.52	**0.13**	**91.69**	0.28	80.6

Here and in the forthcoming tables, the best results are boldfaced and only the audio clips having Type 2 errors are included in the calculation of ER and F1 score. DEW performs best for the ‘glass break’, better for ‘gunshot’, and worst for the ‘baby cry’. This is because, the glass break and gunshot have an impulsive nature [55], and so the rapid transitions in the time domain at their onset were well captured by the wavelet MRA, which has already proven its effectiveness in capturing the transitions due to anomaly in electrocardiogram (ECG) signals [65], financial data [48] and earthquake data [48]. It is concluded from Table 5 that our proposed algorithm is not very effective at the 3^rd^ level for the ‘baby cry’ class.

***Case 3*:** Evaluation of DEW at 5^th^ level

Table 6 shows the performance of DEW for the three classes at the 5^th^ decomposed level.

**Table 6:** 5^th^ level results.

Background	Baby cry		Glass break		Gun shot	
	ER	F1 (%)	ER	F1 (%)	ER	F1 (%)
**Beach**	0.375	74.28	0.1	92.86	0.53	60.87
**Bus**	0.34	75	0.06	96.56	0.23	86.49
**Café**	0.56	47.06	0.2	84.21	0.57	60
**Car**	0.34	77.78	0	100	0.47	73.34
**City-Centre**	0.35	78.78	0.2	80	0.07	94.74
**Forest-Path**	0.44	53.34	0.125	75	0.47	63.16
**Grocery-Store**	0.18	83.34	0.2	66.67	0.4	66.67
**Home**	0.42	55.56	0	100	0.3	72.73
**Library**	0.46	70	0	100	0.19	85.71
**Metro-Station**	0.36	75	0.06	95.24	0.25	75
**Office**	0.4	71.43	0.11	91.67	0.5	64.52
**Park**	0.45	66.67	0.06	95.65	0.21	82.35
**Residential-Area**	0.5	58.34	0	100	0.5	69.57
**Train**	0.47	69.23	0.25	84.85	0.25	83.87
**Tram**	0.6	57.14	0.09	94.74	0.21	86.96
**Average**	0.42	67.53	**0.097**	**90.5**	0.34	75.06

Expect the ER of glass break, there is a significant drop in performance for all the classes at the 5^th^ level when compared to the 3^rd^ level. The underlying reason is the presence of more audio clips with Type 2 errors at the 5^th^ level, which has resulted in more events being undetected by the system and consequently generating more false negatives (FNs), causing higher ER values and lower F1 scores.

***Case 4*:** *Combination of 3^rd^ and 5^th^ levels*

The list of candidate chunks *P* from both the 3^rd^ and 5^th^ levels are now merged as shown in Figs 4 and 5 and all of these chunks are checked for the presence or otherwise of the rare event. The results of different classes in different backgrounds are shown in Table 7.

**Table 7:** 3^rd^ and 5^th^ level combination.

**Background**	**Baby cry**		**Glass break**		**Gun shot**	
	**ER**	**F1 (%)**	**ER**	**F1 (%)**	**ER**	**F1 (%)**
**Beach**	0.25	85.7	0	100	0.35	78.57
**Bus**	0.17	90.3	0	100	0.18	90.48
**Café**	0.56	60.87	0.13	92.31	0.14	92.3
**Car**	0.34	80	0	100	0.23	88.24
**City-Centre**	0.35	80	0.13	92.86	0.14	90.91
**Forest-Path**	0.375	75	0.5	78.95	0.67	64.28
**Grocery-Store**	0.36	75	0.1	94.7	0.53	63.64
**Home**	0.37	66.67	0.09	66.7	0.3	75
**Library**	0.23	85.71	0.05	96.55	0.18	87
**Metro-Station**	0.27	82.35	0.16	90.32	0.25	84.6
**Office**	0.35	75.86	0.05	97.14	0.36	76.5
**Park**	0.5	70.59	0.06	96.55	0.28	83.34
**Residential-Area**	0.35	75.86	0	100	0.28	83.334
**Train**	0.41	74.07	0.1	94.74	0.25	85.7
**Tram**	0.53	63.64	0	100	0.28	84.6
**Average**	0.36	76.11	**0.09**	**93.4**	0.29	82

The combination of the 3^rd^ and 5^th^ levels causes an average improvement in results for all classes when compared to the cases where the 3^rd^ or 5^th^ level is applied alone. The 5^th^ level, although a weak event detector itself, helps strengthen the 3^rd^ level by detecting the events missed by it, at extremely low EBR values, as shown in Table 7.

In the next experiment, the performance of DEW is compared with other baseline systems trained and evaluated on the DCASE 2017 dataset.

***Case 5*:** Comparison of DEW with other state-of-the-art models

The comparison of DEW with other SED algorithms, described in subsection ‘comparison algorithms’, is given in Table 8. All comparison algorithms have been trained and tested on the dataset used by our proposed model. So, results are directly reported from their papers.

**Table 8:** Comparison of ER, F1 score and computational complexity of different SED algorithms.

Algo.	DNN	Av. ER	Av. F1 (%)	Total chunks/ 30 sec clip	Total network learnable parameters	Training epochs	Concatenation with previous frames required?	Additional data samples created for training
[55].	CNN+LSTM	**0.13**	**93**	2583	6200K	10	✓	60,000
[5].	CRNN	0.17	*	2583	756K	200	✓	4767
[57]	CNN & DNN	0.22	88.2	2583	2100K	5	✓	None
[58]	CNN	0.22	88.5	2583	108K	600	✓	5246
[60]	CRNN	**0.13**	**93**	2583	756K	210	✓	9000
DEW	Wavelets	0.24	84	**7.2**	**0**	**1**	**×**	**None**

* NA (Not Available).

As clear from Table 8, our proposed system DEW fails to surpass all the models in terms of F1 score and the models in [5,55,60] in terms of ER. However, its ER is comparable to the models [57,58]. The best performer in terms of ER and F1-score is [55], and our proposed algorithm lags it by 0.11 and 9% respectively. The reasons for falling behind other models may be: 1) due to failure of the detection system, and 2) due to failure of the classification system. These reasons will be investigated in depth in the next section. However, it outperforms all other models in terms of computational resources and the amount of data required for the model training. All the baseline models listed in Table 8 are using DNNs, thus requiring more training data, more computational resources, and more computational time to run multiple epochs on the training dataset. The detection procedure in all models requires checking almost 360 (= 2583/7.2) times more chunks than those required for DEW. As DEW is not a DNN-based architecture, it is computationally fast, and there are no learnable parameters (weights and biases), so it can be implemented on resource-limited lightweight devices. Also, as DEW is using an SVM classifier, it requires very little data for training as compared to the models using deep learning classifiers. All baseline models have reported the use of GPU for training their networks, while DEW does not require any GPU. To detect the anomaly, all algorithms require concatenation of the current frame with previous frames to determine precisely the point of onset, while DEW does not need any concatenation with the previous data to determine the onset time of a rare event. Except the model [57], which does not require additional data creation, all competitive networks have been trained by creating additional training data by utilizing the synthesizer provided by DCASE 2017 [60]. It has already been mentioned that DEW does not even require the data given by DCASE for training purposes except for 1s chunks required for training its SVM classifier. It would be interesting to compare the performance of all algorithms on the same processor and on the same amount of data to highlight the true benefits of DEW.

## Discussion

**Fig 6:**
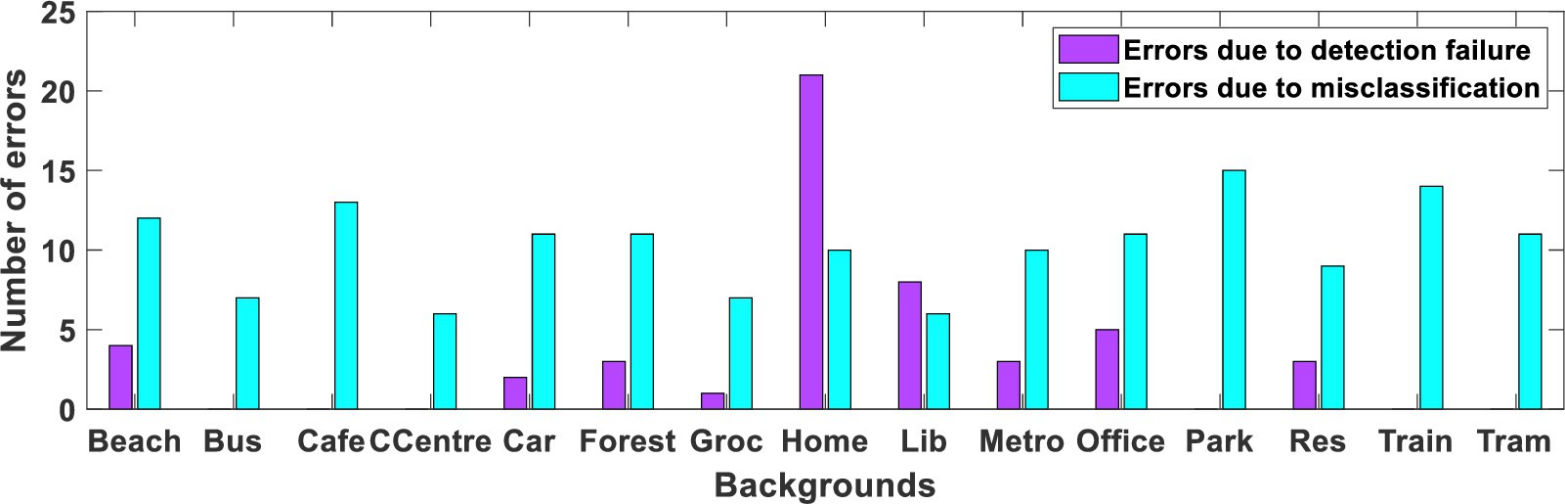
Number of detection and misclassification errors for each type of background.

Our best-performing DEW system (where the 3^rd^ and 5^th^ levels have been combined) is now analyzed to find the main reason for falling behind other SED systems listed in Table 8. There are two types of errors: 1) detection failure and 2) classification failure. The results for different backgrounds are shown in Fig 6.

Of the total 24% ER, 6% is contributed due to detection failures (Type 1 and Type 2 errors), and the remaining 18% is caused by misclassification by the SVM network. Networks like logistic regression, SVMs, and naive Bayes will generalize well to small quantities of data [66]. The reason for the high ER in DEW due to ‘misclassification’ needs further investigation, but it may be due to the inclusion of background in the training dataset of an SVM classifier. The background is included in the dataset of ‘rare event’ classes due to the duration of an event lesser than the one-second interval in the original clip from where the event is extracted. Also, the background varies in different samples of the same rare event class. The duration of the rare event is variable in the audio clips of the DCASE dataset (as given in Table 1), but those variable length chunks of rare events are not suitable for the SVM training. But as these training chunks are extracted from the given audio files of rare events, the inclusion of background in a 1-sec extracted rare event’s chunk is unavoidable in all those cases, where the duration of the rare event is shorter, hence reducing the classifier’s ability to differentiate between the rare event and background effectively. To support this argument, an experiment is performed next, where the duration of chunks given as an input to an SVM classifier is changed and its effect on the accuracy of classification for different classes is observed.

***Case 6*:** Effect of chunk’s duration on SVM accuracy

In this experiment, the effect of changing the duration of extracted chunks on the SVM output is observed. Accuracy (in percentage (%)) is used as a metric for this purpose. Till now, accuracy has been the primary metric for assessing the performance of any classifier. Among many available metrics (e.g. kappa statistic, F-measure, mean absolute error, root mean square error, the area under the precision-recall curve, and the area under the receiver operating curve), the SVM classifier achieves better performance on the accuracy metric than on other metrics [67]. The accuracy is checked for 1, 1.5, and 2s duration and the results are averaged for different classes over all the 15 types of background in Table 9. As the WSN of [61], does not support the size (in terms of the number of samples) of a chunk smaller than the sampling frequency, it is not possible to see the effect of reducing the chunk duration below 1s.

**Table 9:** SVM classifier accuracy for different durations of chunks.

Average accuracy in (%)					
Duration (sec)	Baby cry	Glass break	Gun Shot	Background	Overall System
**1**	**87.5**	81.3	77.9	93.9	85.6
**1.5**	86. 1	**82.5**	**80.6**	**97**	**86.7**
**2**	86.9	78.7	78.2	96.4	85

As clear from the table, the classifier’s accuracy for the class ’baby cry’ is highest for chunks of 1-sec duration, while the other classes exhibit higher accuracy at 1.5s. The classifier’s overall accuracy also improves by increasing chunk duration from 1s to 1.5s. The improvement in the system’s overall accuracy is mainly contributed by the improvement in accuracy of the ‘background’ class, which has no traces of other classes in its training dataset, supporting the postulate that the cleaner chunks of other classes too would have a positive impact on accuracy. Replacing the samples of the included background with zeros in the chunks of rare event classes would create silent zones in these clips. Filtering them out would also modify the features of rare events embedded in the clip.

## Conclusion

Rare event detection by sound is needed in situations where the event detection by camera is not fully effective or too costly. In this paper, a novel wavelet-based approach for rare event detection and classification is proposed by using only the audio recordings. The proposed model is computationally inexpensive in terms of the number of epochs and the candidate chunks that are required to be checked for the presence of rare events. Compared to other deep learning-based competitive networks, our proposed system has no learnable parameters, so it adheres well to real-time needs and can be easily implemented on the lightweight devices. It is anticipated that the availability of purified training data, without any portion of background noise in it, for training the SVM classifier and the proposed model’s integration with VED systems may improve the performance further.
